# Course and predictors of posttraumatic stress-related symptoms among family members of deceased ICU patients during the first year of bereavement

**DOI:** 10.1186/s13054-021-03719-x

**Published:** 2021-08-05

**Authors:** Siew Tzuh Tang, Chung-Chi Huang, Tsung-Hui Hu, Wen-Chi Chou, Li-Pang Chuang, Ming Chu Chiang

**Affiliations:** 1 School of Nursing, Medical College, Chang Gung University, 259 Wen-Hwa 1st Road, Kwei-Shan, Tao-Yuan, 333 Taiwan, R.O.C.; 2 Division of Hematology-Oncology, Chang Gung Memorial Hospital at Linkou, Tao-Yuan, Taiwan, R.O.C.; 3 Department of Nursing, Chang Gung Memorial Hospital at Kaohsiung, Tao-Yuan, Taiwan, R.O.C.; 4 Division of Pulmonary and Critical Care Medicine, Department of Internal Medicine, Chang Gung Memorial Hospital at Linkou, Tao-Yuan, Taiwan, R.O.C.; 5 Department of Respiratory Therapy, Chang Gung University, Tao-Yuan, Taiwan, R.O.C.; 6 Division of Hepato-Gastroenterology, Department of Internal Medicine, Chang Gung Memorial Hospital at Kaohsiung, Kaohsiung, Taiwan, R.O.C.; 7 College of Medicine, Chang Gung University, Tao-Yuan, Taiwan, R.O.C.

**Keywords:** End-of-life care quality, Family satisfaction, PTSD symptoms, Critical illness

## Abstract

**Background/Objective:**

Death in intensive care units (ICUs) may increase bereaved family members’ risk for posttraumatic stress disorder (PTSD). However, posttraumatic stress-related symptoms (hereafter as PTSD symptoms) and their precipitating factors were seldom examined among bereaved family members and primarily focused on associations between PTSD symptoms and patient/family characteristics. We aimed to investigate the course and predictors of clinically significant PTSD symptoms among family members of deceased ICU patients by focusing on modifiable quality indicators for end-of-life ICU care.

**Method:**

In this longitudinal observational study, 319 family members of deceased ICU patients were consecutively recruited from medical ICUs from two Taiwanese medical centers. PTSD symptoms were assessed at 1, 3, 6, and 13 months post-loss using the Impact of Event Scale-Revised (IES-R). Family satisfaction with end-of-life care in ICUs was assessed at 1 month post-loss. End-of-life care received in ICUs was documented over the patient’s ICU stay. Predictors for developing clinically significant PTSD symptoms (IES-R score ≥ 33) were identified by multivariate logistic regression with generalized estimating equation modeling.

**Results:**

The prevalence of clinically significant PTSD symptoms decreased significantly over time (from 11.0% at 1 month to 1.6% at 13 months post-loss). Longer ICU stays (adjusted odds ratio [95% confidence interval] = 1.036 [1.006, 1.066]), financial insufficiency (3.166 [1.159, 8.647]), and reported use of pain medications (3.408 [1.230, 9.441]) by family members were associated with a higher likelihood of clinically significant PTSD symptoms among family members during bereavement. Stronger perceived social support (0.937 [0.911, 0.965]) and having a Do-Not-Resuscitate (DNR) order issued before the patient’s death (0.073 [0.011, 0.490]) were associated with a lower likelihood of clinically significant PTSD symptoms. No significant association was observed for family members’ satisfaction with end-of-life care (0.988 [0.944, 1.034]) or decision-making in ICUs (0.980 [0.944, 1.018]).

**Conclusions:**

The likelihood of clinically significant PTSD symptoms among family members decreased significantly over the first bereavement year and was lower when a DNR order was issued before death. Enhancing social support and facilitating a DNR order may reduce the trauma of ICU death of a beloved for family members at risk for developing clinically significant PTSD symptoms.

**Supplementary Information:**

The online version contains supplementary material available at 10.1186/s13054-021-03719-x.

## Introduction

Intensive care has grown substantially over the past decades worldwide [1–3] to be one of the most resource-intensive acute hospital services [4]. Heavy utilization of the intensive care unit (ICU) over the disease course contributes to high costs of health care [1] and raises concern over optimal use of ICU resources [5], especially for end-of-life (EOL) care, which has increased in the last decade [6]. Landmark studies highlighted the improving but still poor quality of EOL care in ICUs [7–9]. Thus, improving EOL-care quality in ICUs is essential [10] for improving the quality of death and dying [11], facilitating bereavement adjustment among family members [10], and counteracting unsustainable ICU care expenditures [10, 11].

Family members of ICU patients are an integral part of ICU care and a critical target for improving EOL-care quality in ICUs [12, 13]. The uncertain trajectory of critical illnesses, the frightening nature of aggressive life-prolonging treatments [14], and the beloved’s unexpected and eventual death are traumatic events for family members of ICU patients [15]. Therefore, family members are at increased risk for post-intensive care syndrome (PICS-F)—new or worsening impairments in physical, cognitive, or mental health status arising after a beloved’s critical illness and persisting beyond acute care hospitalization [16], including posttraumatic stress disorder (PTSD) [14]. PTSD takes a toll on physical [17, 18] and mental [17–19] health, personal relationship/social functioning [20] and poses a considerable economic burden for individuals, health care systems, and societies [21, 22]. These striking characteristics make PTSD a public mental health priority [19].

A better understanding of the modifiable risk factors for developing posttraumatic stress-related symptoms (hereafter as PTSD symptoms) may allow health care professionals to preemptively identify family members who are vulnerable to PTSD symptoms during bereavement and develop actionable high-quality, family-centered EOL care in ICUs. However, most studies [14] that examined potential predictors of PTSD symptoms among family members of ICU patients were done primarily on those of ICU survivors [9, 14, 23–27], while only a handful were done on those of deceased patients [7–9, 28–34]. Furthermore, only a few of these studies found significant associations, and the majority of them focused on immutable family and patient characteristics [7, 9, 14, 23–26, 28, 29]. From the few [28, 29, 33] that examined process-based quality indicators of EOL care in ICUs, it was found that participation in early family meetings and presence at the time of patient’s death were associated with worse PTSD symptoms among family members [28], whereas withdrawal of life-sustaining treatments (LSTs) had no association [29, 33]. The association between family’s perception/satisfaction of the quality of death in ICUs and PTSD was inconclusive [8, 9, 25, 26, 30].

Studies on PTSD among family members of ICU patients [7–9, 23–34] also remain limited by methodological insufficiencies. First, most studies were cross-sectional [7–9, 23, 25, 27–29, 32], with PTSD symptoms measured across a wide range of time (1–9 months post-loss), which makes it difficult to compare prevalence of PTSD across studies and precludes the possibility of establishing temporal associations with predictors. Second, while most of the six longitudinal studies [24, 26, 30, 31, 33, 34] assessed PTSD symptoms at 3 [30, 31, 33, 34] and 6 [26, 30, 31, 33, 34] months post-loss, only few studies had data at 1–2 [24, 26] and 12 [30, 31, 33] months post-loss. Third, sample sizes tended to be small (*N* = 30–475 and five [8, 23, 25, 26, 29] out of 15 studies had < 100 subjects), limiting the power to detect potential predictors of PTSD symptoms. Finally, none were from Asian countries which may have different cultural reactions toward grief and only one study accounted for family members’ preexisting mental health and medical conditions [7]. Thus, this study aimed to comprehensively examine the course and predictors of PTSD over the first bereavement year among a large cohort of Taiwanese family members whose beloved died in the ICU. This study specifically focused on investigating the modifiable factors that contribute to EOL-care quality in ICUs.

## Materials and methods

### Study design and setting

In this prospective, longitudinal observational study, we extended our previous studies that assessed process-based quality of EOL care in ICUs [35] and associations between the quality indicators of EOL care in ICUs and bereaved family members’ anxiety and depressive symptoms [36] to a larger sample size and examined associations of EOL-care quality in ICUs with development of PTSD symptoms among bereaved family members. Patients and their primary family members were consecutively recruited from January 2018 to January 2020 and followed through December 2020 from two academically affiliated level III medical ICUs in Taiwan (223 and 201 beds, respectively). All study ICUs were staffed by intensivists, had a 1:2 nurse-to-patient ratio per shift, and implemented an open visiting policy. Palliative care, a do-not-resuscitate (DNR) order, and formal family meetings for dying patients were not mandated but were promoted by the Taiwanese government. Bed-side physician-family prognostic and EOL-care discussions were commonly conducted.

### Sampling strategy and study participants

Eligible ICU patients were identified as those at high risk of death, i.e., had an Acute Physiology and Chronic Health Evaluation (APACHE) II score ≥ 20. Patients who died within 3 days of ICU admission were excluded, as time did not allow for the implementation of high-quality EOL care [8, 9, 25, 30]. Eligible adult family members were those who self-identified as having legal authority to act as a surrogate for their beloved’s medical decisions. This study was approved (201700343B0). Each patient’s legal family surrogate signed informed consent for their participation and for reviewing the patient’s medical record.

### Measures

#### Outcome variable

*Posttraumatic stress-related symptoms (PTSD symptoms)* of bereaved family members were measured by the Impact of Event Scale-Revised (IES-R), a 22-item questionnaire with established test–retest reliability, internal consistency, and concurrent validity for ICU patients’ family members [37, 38]. The IES-R cannot diagnose PTSD but acts as a screening instrument for PTSD symptoms and is most widely used with ICU family members [8, 9, 23–27, 29–34] as indicated by a systematic review [39]. The IES-R has three subscales: intrusion, avoidance, and hyperarousal. Each item is rated for its distress level during the past week on a Likert scale from 0 (not at all) to 4 (extremely). IES-R scores ≥ 33 indicate clinically significant likelihood of PTSD, with a sensitivity of 0.91 and specificity of 0.82 [37]. Internal consistency (Cronbach’s α) and test–retest reliability of the IES-R ranged 0.925–0.953 and 0.693–0.868, respectively, over the first bereavement year.

#### Independent variables

*Chart-derived, process-based indicators of high-quality EOL care in ICUs* [13] were selected to focus on communication with and psychosocial support for family members in order to facilitate informed EOL-care decision-making and limit potentially inappropriate LSTs. These indicators included physician-family prognostic communication, family meetings conducted, palliative care provided, social worker involvement, a DNR issued prior to death, death without cardiopulmonary resuscitation (CPR), withdrawal of any LSTs, and family presence at death [13, 35].

*Family satisfaction with EOL care in ICUs* was evaluated by the Family Satisfaction in the Intensive Care Unit questionnaire (FS-ICU) [40, 41]. The FS-ICU Care subscale (14 items) assesses (1) satisfaction with information access, (2) care quality, continuity, and accessibility, and (3) ICU and waiting room atmosphere. The FS-ICU Decision-Making subscale (10 items) measures family satisfaction with the content, completeness, and consistency of information received, as well as the amount and quality of support received during the decision-making process. Item responses were rescaled from the original 1–5 Likert scale to a scale from 0 (least satisfied) to 100 (most satisfied). Two items were removed from the total FS-ICU Care score as they were rated as “not applicable” by > 20% of family participants [41] (agitation management, 31.29% and waiting room atmosphere, 56.32%).

*Patient demographics and clinical characteristics* included gender, age, diagnosis (cancer vs. others), disease severity (APACHE II scores), and ICU length of stay (LOS). We explored comorbidity for patients by examining whether any of 21 common diseases (e.g., cancer, cardiovascular disease, congenital heart failure, chronic obstructive pulmonary disease, etc.) other than the underlying primary disease were recorded in the medical chart. If any diseases other than the underlying primary disease were recorded, we coded the variable of comorbidity as “yes,” otherwise as “no.”

*Family characteristics* included gender, age, relationship to patient (spouse, child, or other), education level (≤ or > junior high school), financial sufficiency to make ends meet (yes *or* no), preexisting mental health or medical conditions, and social support. Preexisting conditions were evaluated by the 7-item Mental Health Questionnaire [7]. Items include the frequency of emergency room visits and hospitalizations, and pain or mood medication use the year before the patients’ critical illness. These questions were re-categorized into “yes” or “no.” Perceived social support was measured by the 19-item Medical Outcomes Study Social Support Survey (MOS-SSS) [42], which assesses emotional, informational, tangible, and affectionate support, as well as positive social interaction. The total score was computed and transformed to range from 0 to 100, with a higher score indicating better perceived support.

### Data collection

Patients’ and family members’ demographics and preexisting comorbidities were recorded at enrollment. Patient disease severity (APACHE II scores) was assessed over the ICU stay. Data for patient-level process-based quality measures over the ICU stay were extracted from medical records by trained, experienced research assistants with adequate inter-rater reliability [35]. Family members’ PTSD symptoms and perceived social support were assessed in telephone interviews by trained, experienced research assistants at 1, 3, 6, and 13 months post-loss. The telephone interview is semi-structured—data collectors were instructed to interview study participants with example scripts but were free to modify the interview scripts based on participants’ needs. To ensure uniformity of data collection, prior to launch of this study, research assistants’ reliability in data collection was confirmed by comparing the data they collected with that recorded by the principal investigator on five pilot cases and among research assistants for inter-rater reliability on another five pilot cases with a minimum of 95% agreement required. To maintain 95% inter-rater reliability, the principal investigator intermittently checked agreement on reliability throughout the study. Phone calls were made during different periods over a week (e.g., morning and evening, different weekdays) if the first attempt failed to reach participants. Telephone interviews were conducted at 1 month post-loss because PTSD symptoms are those that persist ≥ 1 month after traumatic exposure [37], and at 13 months post-loss to avoid measuring the anniversary effect. Family satisfaction with EOL-care quality was assessed 1 month post-loss only.

### Statistical analysis

A multivariate logistic regression analysis with the generalized estimating equation method was used to examine associations between the proposed predictors and clinically significant PTSD symptoms (IES-R score ≥ 33) among family members over their first bereavement year. APACHE II scores and process-based quality measures of EOL care last measured prior to the patient’s death were used for statistical analysis. The regression estimate for each independent variable in the logistic regression models was exponentiated to transform into adjusted odds ratio (AOR) with 95% confidence interval (CI).

## Results

### Participant characteristics

**Fig. 1:**
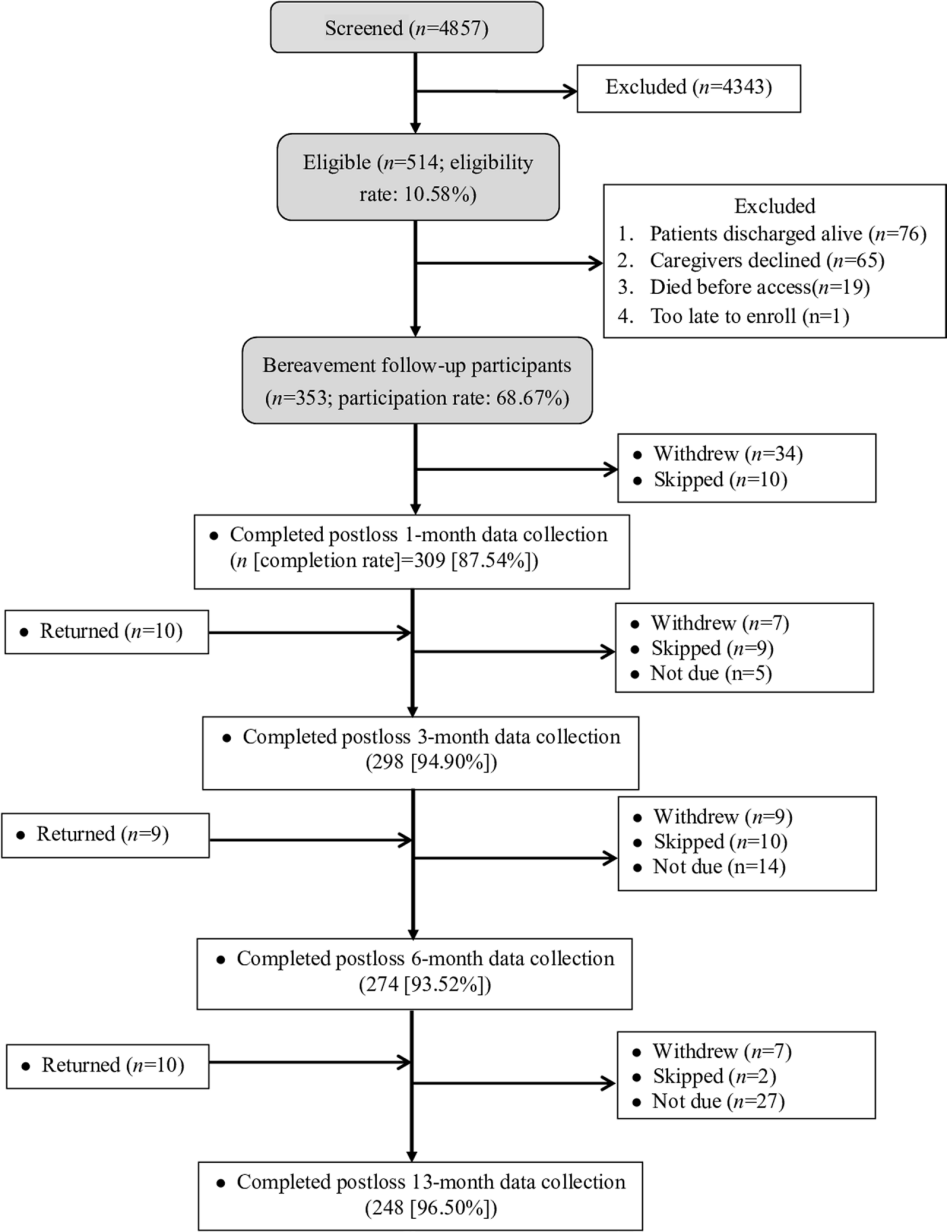
Case flowchart post-loss 13 month

Among the 353 recruited patients (Fig. 1), 319 family members (90.37%) participated in bereavement surveys and constituted the study participants. Bereavement surveys at 1, 3, 6, and 13 months post-loss were completed by 309, 298, 274, and 248 family members, respectively (Fig. 1). Participants and non-participants of bereavement surveys (either declined at the beginning or withdrew participation) did not differ in patient (Additional file 1: Table S1) or family (Additional file 2: Table S2) demographics. Characteristics of the patient and family participants are shown in Tables 1 and 2. The top three underlying diseases among patient participants were cancer (50.2%), pulmonary diseases (6.9%), and cardiovascular diseases (4.7%). Few family participants had preexisting medical conditions. The process-based indicators of high-quality EOL care in ICUs are shown in Table 3. Family satisfaction with the care and decision-making process in ICUs was low (Table 3).

**Table 1:** Characteristics of patient participants (*N* = 319)

Variable	*n*	%
Gender		
Male	203	63.6
Female	116	36.4
Disease		
Cancer	160	50.2
Pulmonary	22	6.9
Cardiovascular	15	4.7
Gastrointestinal	3	0.9
Renal	16	5.0
Other	103	32.3
Acute symptoms/problems on admission		
Respiratory failure/distress	166	52.0
Infection	90	28.2
Shock	24	7.5
Bleeding	10	3.1
Cardiac arrest	12	3.8
Others	17	5.3
Comorbidity		
Yes	271	85.0
No	48	15.0

Variable	Mean (SD)	Range	Median
Age (years)	66.67 (14.22)	22–101	67.0
APACHE^a^	28.37 (5.37)	8–45	28.0
SOFA^a^	12.32 (4.03)	1–22	12.0
Length of ICU stay (days)	21.18 (15.09)	3–106	17.0
Time from ICU admission to enrollment (days)	14.48 (12.60)	3–77	10.0
Time from enrollment to death (days)	7.27 (8.35)	1–58	4.0

^a^Measures of disease severity at enrollment

*APACHE* Acute Physiology and Chronic Health Evaluation, *SOFA* sequential organ failure assessment

**Table 2:** Characteristics of family participants (*N* = 319)

Variable		*n*	%
Gender			
Male		130	40.75
Female		189	59.2
Relationship with the patient			
Spouse		94	29.5
Adult child		173	54.2
Others		52	16.3
Age (years)			
Mean (SD)	49.86 (12.53)		
Range: 21–80	Median: 50.0		
Marital status			
Single		68	21.3
Married		241	75.5
Separated		10	3.1
Educational level			
> high school		158	49.5
≤ high school		161	50.5
Financial sufficiency to make ends meet (*n* = 312)			
Yes		268	85.9
No		44	14.1
*Preexisting mental health and medical problems in the past year*			
Hospitalization for medical problems			
Yes		14	4.4
No		305	95.6
Emergency room visit			
Yes		22	6.9
No		297	93.1
Medication use for anxiety			
Yes		8	2.5
No		311	97.5
Hospitalization for mental health problems			
Yes		0	0.0
No		319	100.0
Pain medication use			
Yes		35	11.0
No		284	89.0
Medication use for depression or other psychiatric disturbances			
Yes		3	0.9
No		316	99.1

**Table 3:** Quality of end-of-life care identified on the medical records of patients who died in intensive care units (*N* = 319)

Quality indicator	Prevalence	
	*n*	%
Physician-family prognostic communication	288	90.28
Family meetings conducted	68	21.32
Palliative care provided	235	73.67
Social worker involvement	27	8.46
Do-not-resuscitate order issued	307	96.27
Death without cardiopulmonary resuscitation	299	93.73
Withdrawal of life-sustaining treatments^a^	66	20.69
Death with full life support^b^	253	79.31
Family presence at patient’s death	245	76.90
Family satisfaction with EOL care in ICUs^c^	Mean	SD
FS-ICU Care subscale	67.14	16.30
FS-ICU Decision-Making subscale	76.20	14.76

^a^Life-sustaining treatments included intubation with mechanical ventilation support, vasopressors, hemodialysis, enteral and/or parenteral hydration and nutrition, antibiotics, and transfusion of blood products

^b^Without orders to withdraw any life support

^c^Measured by the Family Satisfaction in the Intensive Care Unit questionnaire (FS-ICU)

### Predictors of clinically significant PTSD symptoms among family members in the first bereavement year

Clinically significant PTSD symptoms were found in 11.0%, 3.7%, 1.1%, and 1.6% of bereaved family members at 1, 3, 6, and 13 months post-loss, respectively. The likelihood of having clinically significant PTSD symptoms was lower at 3 months (AOR [95% CI] = 0.062 [0.016, 0.244]), 6 months (0.052 [0.018, 0.146]), and 13 months (0.214 [0.106. 0.431]) than at 1 month post-loss (Table 4).

**Table 4:** Predictors of PTSD symptoms over the first year of bereavement (*N* = 319)

	AOR	95% CI		*p*
*Time since a beloved’s death (months)*				
1	Ref			
3	0.062	0.016	0.244	< .0001
6	0.052	0.018	0.146	< .0001
13	0.214	0.106	0.431	< .0001
*Patient characteristics*				
Age (years)	0.980	0.924	1.040	0.510
Gender (female vs. male)	2.867	0.884	9.293	0.079
Diagnosis (cancer vs. non-cancer)	0.377	0.124	1.151	0.087
APACHE II score^a^	0.976	0.905	1.052	0.530
ICU length of stay (days)	1.036	1.006	1.066	0.019
*Family characteristics*				
Age (years)	0.978	0.906	1.055	0.564
Gender (female vs. male)	2.644	0.834	8.380	0.099
Relationship with the patient (vs. Others)				
Spouse	0.940	0.245	3.598	0.927
Adult child	0.230	0.024	2.238	0.205
Marital status (married vs. unmarried)	1.963	0.515	7.486	0.323
Educational attainment (> vs. ≤ junior high school)	1.582	0.471	5.307	0.458
Financial insufficiency to make ends meet (yes vs. no)	3.166	1.159	8.647	0.025
*Preexisting physical and mental health problems in family members*^b^				
Hospital admission with physical problems	1.156	0.123	10.853	0.899
Emergency room visits	3.395	0.773	14.908	0.106
Pain medication use	3.408	1.230	9.441	0.018
Mood medication use	0.534	0.052	5.526	0.599
*Family’s perceived social support*^c^	0.937	0.911	0.965	< .0001
*Process-based indicators of high-quality end-of-life care*^d^				
Physician-family prognostic communication	1.686	0.225	12.666	0.612
Family meetings conducted	1.022	0.357	2.926	0.968
Palliative care provided	1.549	0.443	5.409	0.493
Social worker involvement	0.501	0.111	2.273	0.371
Do-not-resuscitate order issued	0.073	0.011	0.490	0.007
Death without cardiopulmonary resuscitation	10.871	0.343	344.089	0.176
Withdrawal of life-sustaining treatments^e^	0.977	0.374	2.551	0.961
Family presence at patient’s death	2.247	0.752	6.707	0.147
*Family satisfaction with end-of-life care in ICUs*^f^				
Satisfaction with ICU care	0.988	0.944	1.034	0.604
Satisfaction with decision-making	0.980	0.944	1.018	0.310

^a^APACHE: Acute Physiology and Chronic Health Evaluation

^b^Reference for each preexisting physical and mental health problem: Not experiencing the problem

^c^Measured by the Medical Outcomes Study Social Support Survey

^d^Reference for each quality indicator: Not experiencing the event

^e^Life-sustaining treatments included intubation with mechanical ventilation support, vasopressors, hemodialysis, enteral and/or parenteral hydration and nutrition, antibiotics, transfusion of blood products

^f^Measured by the Family Satisfaction in the Intensive Care Unit questionnaire

Among patient characteristics, only ICU LOS was associated with clinically significant PTSD symptoms among family members (AOR increased by 1.036 [95% CI = 1.006, 1.066] with each additional day in the ICU). Among the family member characteristics, only financial insufficiency and pain medication use within the past year was associated with increased likelihood of clinically significant PTSD symptoms (AOR = 3.166 [1.159, 8.647] and 3.408 [1.230, 9.441], respectively). However, stronger perceived social support was negatively associated with clinically significant PTSD symptoms (AOR reduced by 0.937 [95% CI = 0.911, 0.965] with each unit increase in the MOS-SSS score).

Among the process-based indicators of high-quality EOL care in ICUs, only a DNR order issued before death was associated with a lower likelihood of clinically significant PTSD symptoms (AOR = 0.073 [0.011, 0.490]) (Table 4). Having a higher level of satisfaction with the care or decision-making process in ICUs was associated with a lower likelihood of clinically significant PTSD symptoms, but this was not significant (AOR = 0.988 [0.944, 1.034] and 0.980 [0.944, 1.018], respectively) (Table 4).

## Discussion

In this longitudinal study, the prevalence of clinically significant PTSD symptoms among family members of deceased ICU patients decreased significantly over the first bereavement year (from 11.0% at 1 month to 1.6% at 13 months post-loss). Even though a similar trend was seen in prior studies [30, 31, 33], the observed prevalence of clinically significant PTSD symptoms in our study was substantially lower than that reported in other longitudinal (19–46.2% at 3–12 months post-loss) [30, 31, 33, 34] and cross-sectional studies (14–51% at 3–18 months post-loss) [7, 28, 29, 32]. Of note, the reported prevalence of clinically significant PTSD symptoms measured by self-reported screening instruments (including the IES-R) was 20.2% (95% CI 16.6–24.0%) among adult critical care survivors [43], 3.3–35.1% among burn survivors during hospitalization [44], 7.3% (4.5–11.7%)–13.8% (9.5–19.6%) among cancer survivors [45]. All these populations experience traumatizing diseases and treatments. Our observed prevalence of clinically significant PTSD symptoms was closer to other reports: 3–62% among family caregivers of ICU survivors [14] and 11.6% (9.6–11.6%) among parents of children with cancer [46].

The lower rates of clinically significant PTSD symptoms among the bereaved family caregivers in our study, as compared to those reported in studies from Western countries [7–9, 28–34], may be due to the cultural difference in grief reactions between Taiwanese and Western cultures. In Taiwanese culture, caring for an ill relative is viewed as a natural part of family life based on the concept of filial duty rooted in Confucian doctrines [47]. Family members in Asian cultures, which are more family-oriented, are willing to help each other adjust to the loss of a beloved by providing stronger financial and emotional support [48] than those from Western cultures, which are more individualistic. Thus, bereaved Taiwanese family members’ risk of significant PTSD symptoms may be lower.

The high rate (50.2%) of cancer patients in our study may be another reason for our low prevalence of significant PTSD symptoms. Azoulay et al. report cancer diagnosis as a factor predisposing family members to more severe PTSD symptoms [9]. However, this finding may be confounded by other factors, such as patient’s death in the ICU and the need for family to make EOL-care decisions, which were both associated with higher rates of PTSD [9]. The terminal-illness trajectory for cancer patients is commonly anticipated and predictable than the sudden and unexpected illness trajectory from cardiovascular or pulmonary diseases. In our study, family members of cancer patients had a lower likelihood of significant PTSD symptoms than those of patients with non-cancer diagnoses. However, this did not reach a statistical significance, likely due to the low prevalence of significant PTSD symptoms in our study. The specific characteristics of dying and death from cancer may reduce the overwhelming and uncontrollable trauma due to a beloved’s death.

We intended to focus on associations between quality indicators of EOL care in ICUs and clinically significant PTSD symptoms among family members during the first bereavement year. We did not find many significant associations for either process-based indicators [28] of or family satisfaction with EOL care in ICUs [8, 25, 26], which is consistent with prior studies. We observed low family satisfaction with care (mean [SD] = 67.14 [16.30]) and decision-making process (72.60 [14.76]) in ICUs which may be attributable to different expectations of care in differing health care contexts as reported [49], but further cross-country and -cultural comparisons of SF-ICU are warranted.

However, having a DNR order documented before death was associated with a lower likelihood of clinically significant PTSD symptoms among family members (AOR [95% CI] = 0.073 [0.011, 0.490]). Taiwan’s government has launched multiple nationwide projects to facilitate dissemination of hospice philosophy and palliative care services over the past three decades with the help from non-governmental organizations. The goal of these efforts was to improve the quality of EOL care, with an emphasis on promoting DNR orders and avoidance of LSTs that do not benefit patients at EOL. The Taiwanese government promotes DNR orders by legislation and by educating health care professionals. This facilitates prognostic disclosure and respects patient’s and family’s wishes to forgo LSTs, minimizing suffering and allowing patients to die in peace. Therefore, having a DNR order may help prevent bereaved family members from experiencing a traumatized loss and developing significant PTSD symptoms.

However, despite the high prevalence of having a DNR order documented before death, Taiwanese patients dying in ICUs heavily used life support until death (79.31%) with few decisions to withdraw LSTs (20.69%). This paradox may be attributable to Taiwanese physician and family factors in withdrawing LSTs. ICU physicians in Asia, including in Taiwan, report being less likely than Western physicians to withdraw LSTs, but more likely to “do everything” for a patient with an irreversible condition like severe septic shock [50]. Most importantly, Taiwanese family members have a strong cultural obligation based on the Confucian doctrine of filial duty [47] to use every possible means to keep a beloved alive. Therefore, even if family members were fully informed of their beloved’s poor prognosis and were advised by physicians to withdraw LSTs, they might hesitate to initiate or accept the suggestion, especially for those whom withdrawal is interpreted as inducing immediate death, e.g., mechanical ventilation support or vasopressors. Such physician-family disagreement on the use of LSTs may partially contribute to our observed low satisfaction with care and decision-making in ICUs but warrants further cross-cultural investigation.

ICU length of stay was the only patient factor that was associated with bereaved family members’ likelihood of significant PTSD symptoms. This was contrary to the lack of association previously reported [24]. However, McGiffin and colleagues [15] suggested that if the ICU environment was itself a traumatic stressor, then incremental increases in ICU exposure (e.g., longer ICU stays) may be expected to approximate a dose–response relationship to worse psychological outcomes. Our study provides empirical support to this notion: spending more time in an ICU increased family members’ likelihood of significant PTSD symptoms during the first bereavement year.

We found that reported financial insufficiency increased family member’s risk of significant PTSD symptoms [14, 33], whereas stronger perceived social support decreased the risk. Furthermore, family members who reported receiving medical care in the year prior to the patient’s critical illness were generally more likely to develop significant PTSD symptoms, consistent with a prior study [7]. This trend reached significance in family members who reported using medications for pain, which is dissimilar from Gries and colleagues’ finding [7] that use of medication for mood is positively associated with PTSD symptoms. Our finding may be attributable to the fact that Asian people tend to somatize their emotional distress by reporting pain [51] to avoid stigmatization from psychiatric disorders [52]. Further investigation of PTSD symptoms across cultures and socioeconomic class is needed.

Our study has several limitations. Participants were sampled from only two hospitals in Taiwan, which may limit generalizability of our findings to national/international populations, especially considering cultural variations in grief reactions during bereavement in Western and Eastern countries [47]. Our findings need to be replicated in countries where cultural, societal, and health care characteristics are substantially different. Our results do not apply to family members of patients who died within 3 days of ICU admission, or to those who withdrew from post-loss surveys. PTSD symptoms were measured with the IES-R, which is not diagnostic of PTSD, thereby likely overestimating the prevalence of PTSD. However, this characteristic also helps prevent providers from overlooking the family’s need for psychological support. Our study cannot infer a causal relationship or exclude the possible impact of unmeasured factors, e.g., concordance between family members’ preferred and actual decision-making roles and physician-family agreement on appropriateness of treatment which may be more powerful than the current identified indicators of high-quality EOL care in ICUs in association with bereaved family members’ PTSD symptoms. We did not include anxiety or depressive symptoms as potential predictors but we will explore associations among these three types of psychological distress in a forthcoming study.

## Conclusion and clinical implications

The prevalence of clinically significant PTSD symptoms experienced by family members of deceased ICU patients decreased significantly over the first bereavement year. The risk was lower with stronger perceived social support, when a DNR order was issued before the patient’s death. However, the risk was higher with longer ICU stays and if a family member reported financial insufficiencies or pain medication used the year prior to the patient's critical illness. During EOL care for ICU patients, special attention should be directed to family members at risk for significant PTSD symptoms, especially when ICU stays are longer, or when family members suffer from pain, face financial hardship, or have weak social support. Facilitating a DNR order before the patient’s death not only avoids unnecessary ICU patient suffering [48], but also decreases bereaved family members’ risk of significant PTSD symptoms, thus reducing tremendous costs to individuals [16–20], health care systems, and society [21, 22].

## Supplementary Information

**Additional file 1**. Comparisons of patient characteristics at enrollment.

**Additional file 2**. Family caregiver characteristics at enrollment.

## Data Availability

The sharing of anonymized data from this study is restricted due to ethical and legal constrictions. Data contain sensitive personal health information, which is protected under The Personal Data Protection Act, thus making all data requests subject to Institutional Review Board (IRB) approval. Per Chang Gung Memorial Hospital (CGMH) IRB, the data that support the findings of this study are restricted for transmission to those outside the primary investigative team. Data sharing with investigators outside the team requires IRB approval. All requests for anonymized data will be reviewed by the research team and then submitted to the CGMH IRB for approval.

## Abbreviations

AOR: adjusted odds ratio
APACHE: Acute Physiology and Chronic Health Evaluation
CI: confidence interval
CPR: cardiopulmonary resuscitation
DNR: do not resuscitate
EOL: end of life
FS-ICU: Family Satisfaction in the Intensive Care Unit questionnaire
ICU: intensive care unit
IES-R: Impact of Event Scale-Revised scale
LSTs: life-sustaining treatments
MOS-SSS: Medical Outcomes Study Social Support Survey
PTSD: posttraumatic stress disorder

## Code availability

Specifications for data abstraction from the medical records and SAS codes for statistical analyses are available from the corresponding author upon reasonable request.
