# Supplementary material for: Course and predictors of posttraumatic stress-related symptoms among family members of deceased ICU patients during the first year of bereavement
Source: Crit Care. 2021 Aug 5;25:282. doi: 10.1186/s13054-021-03719-x (PMC8340476; doi:10.1186/s13054-021-03719-x)
Supplement: Supplementary file 1 — Additional file 1. Comparisons of patient characteristics at enrollment. [file 13054_2021_3719_MOESM1_ESM.docx]

**Additional file 1: Table S1. Comparisons of patient characteristics at enrollment (*N* =353)**

| Variable | Participants  (*n* =287) | Rejection for bereavement follow-ups  (*n* =34) | Withdrawal of bereavement follow-ups (n=32) | *P* |
| --- | --- | --- | --- | --- |
| Gender, *n* (%) |  |  |  | .592 |
| Male | 181 (63.1%) | 24 (70.6%) | 22 (68.8%) |  |
| Female | 106 (36.9%) | 10 (29.4%) | 10 (31.2%) |  |
| Disease, *n* (%) |  |  |  | .597 |
| Cancer | 143 (49.8%) | 20 (58.8%) | 17 (53.1%) |  |
| Chest | 21 (7.3%) | 2 (5.9%) | 1 (3.1%) |  |
| Cardiovascular | 14 (4.9%) | 2 (5.9%) | 1 (3.1%) |  |
| Digestive | 3 (1.0%) | 0 (0.0%) | 0 (0.0%) |  |
| Kidney | 15 (5.3%) | 2 (5.9%) | 1 (3.1%) |  |
| Other | 91 (31.7%) | 8 (23.5%) | 12 (37.5%) |  |
| Acute symptoms/problems at admission, *n* (%) | | | | .865 |
| Respiratory failure/distress | 149 (51.9%) | 17 (50.0%) | 17 (53.1%) |  |
| Infection | 81 (28.2%) | 9 (26.5%) | 9 (28.1%) |  |
| Shock | 23 (8.0%) | 1 (2.9%) | 1 (3.1%) |  |
| Bleeding | 9 (3.1%) | 2 (5.9%) | 1 (3.1%) |  |
| Cardiac arrest | 10 (3.5%) | 0 (0.0%) | 2 (6.3%) |  |
| Other | 15 (5.2%) | 5 (14.7%) | 2 (6.3%) |  |
| Chronic disease , *n* (%) |  |  |  | .723 |
| Yes | 245 (85.4%) | 30 (88.2%) | 26 (81.3%) |  |
| No | 42 (14.6%) | 4 (11.8%) | 6 (18.8%) |  |
| Age, mean (SD) | 66.57 (14.46) | 66.03 (10.70) | 67.53 (12.05) | .905 |
| APACHE, mean (SD) | 28.34 (5.42) | 28.18 (5.92) | 28.66 (4.98) | .934 |
| SOFA, mean (SD) | 12.42 (4.10) | 12.71 (4.10) | 11.44 (3.27) | .372 |

APACHE: Acute Physiology and Chronic Health Evaluation; SOFA: Sequential Organ Failure Assessment
