# Supplementary material for: Course and predictors of posttraumatic stress-related symptoms among family members of deceased ICU patients during the first year of bereavement
Source: Crit Care. 2021 Aug 5;25:282. doi: 10.1186/s13054-021-03719-x (PMC8340476; doi:10.1186/s13054-021-03719-x)
Supplement: Supplementary file 2 — Additional file 2. Family caregiver characteristics at enrollment. [file 13054_2021_3719_MOESM2_ESM.docx]

**Additional file 2: Table S2.** **Family caregiver characteristics at enrollment (*N* = 353)**

| Variable | Participants  (*n* =287) | Rejection for bereavement follow-ups  (*n* =34) | Withdrawal of bereavement follow-ups (n=32) | *P* |
| --- | --- | --- | --- | --- |
| Age, *n*. (%) |  |  |  | .029 |
| 21-45 | 116 (40.4%) | 19 (55.9%) | 9 (28.1%) |  |
| 46-55 | 79 (27.5%) | 10 (29.4%) | 9 (28.1%) |  |
| 56-65 | 55 (19.2%) | 4 (11.8%) | 11 (34.4%) |  |
| >65 | 37 (12.9%) | 1 (2.9%) | 3 (9.4%) |  |
| Gender, *n* (%) |  |  |  | .029 |
| Male | 124 (43.2%) | 14 (41.2%) | 6 (18.8%) |  |
| Female | 163 (56.8%) | 20 (58.8%) | 26 (81.3%) |  |
| Marital status, *n* (%) |  |  |  | .109 |
| Single | 64 (22.3%) | 11 (32.4%) | 4 (12.5%) |  |
| Married/Cohabiting | 216 (75.3%) | 21 (61.8%) | 25 (78.1%) |  |
| Separated/Widowed | 7 (2.4%) | 2 (5.9%) | 3 (9.4%) |  |
| Educational level, *n* (%) |  |  |  | .133 |
| >High school | 142 (49.5%) | 23 (67.6%) | 16 (50.0%) |  |
| ≦High school | 145 (50.5%) | 11 (32.4%) | 16 (50.0%) |  |
| Financial status, *n* (%) |  |  |  | .103 |
| Making ends meet | 244 (85.0%) | 25 (73.5%) | 24 (75.0%) |  |
| Financial strain | 37 (12.9%) | 7 (20.6%) | 7 (21.9%) |  |
| Other | 6 (2.1%) | 2 (5.9%) | 1 (3.1%) |  |
| Relationship, *n* (%) |  |  |  | .575 |
| Spouse | 83 (28.9%) | 7 (20.6%) | 11 (34.4%) |  |
| Child | 155 (54.0%) | 25 (73.5%) | 18 (56.3%) |  |
| Other | 49 (17.1%) | 2 (5.9%) | 3 (9.4%) |  |
| Chronic disease, *n* (%) |  |  |  | .225 |
| Yes | 100 (34.8%) | 7 (20.6%) | 12 (37.5%) |  |
| No | 187 (65.2%) | 27 (79.4%) | 20 (62.5%) |  |
| Living with the patient, *n* (%) | |  |  | .888 |
| Yes | 191 (66.6%) | 22 (64.7%) | 20 (62.5%) |  |
| No | 96 (33.4%) | 12 (35.3%) | 12 (37.5%) |  |

| Variable | Participants  (*n* =287) | Rejection for bereavement follow-ups  (*n* =34) | Withdrawal of bereavement follow-ups (n=32) | *P* |
| --- | --- | --- | --- | --- |
| Hospitalization for mental health problems, *n* (%) | | | | 1.000 |
| Yes | 0 (0.0%) | 0 (0.0%) | 0 (0.0%) |  |
| No | 287 (100.0%) | 34 (100.0%) | 32 (100.0%) |  |
| Hospitalization for medical problems, *n* (%) | | | | .428 |
| Yes | 13 (4.5%) | 0 (0.0%) | 1 (3.1%) |  |
| No | 274 (95.5%) | 34 (100.0%) | 31 (96.9%) |  |
| Emergency room visit, *n* (%) | |  |  | .667 |
| Yes | 20 (7.0%) | 1 (2.9%) | 2 (6.3%) |  |
| No | 267 (93.0%) | 33 (97.1%) | 30 (93.8%) |  |
| Medication use for pain problems, *n* (%) | | |  | .676 |
| Yes | 33 (11.5%) | 3 (8.8%) | 2 (6.3%) |  |
| No | 254 (88.5%) | 31 (91.2%) | 30 (93.8%) |  |
| Medication use for anxiety problems, *n* (%) | | |  | .628 |
| Yes | 7 (2.4%) | 0 (0.0%) | 1 (3.1%) |  |
| No | 280 (97.6%) | 34 (100.0%) | 31 (96.9%) |  |
| Medication use for depressive problems or other psychiatric disturbances, *n* (%) | | | | .707 |
| Yes | 3 (1.0%) | 0 (0.0%) | 0 (0.0%) |  |
| No | 284 (99.0%) | 34 (100.0%) | 32 (100.0%) |  |
